# Mapping the health management journey of allogeneic hematopoietic stem cell transplantation: a qualitative study

**DOI:** 10.1186/s12913-026-15348-7

**Published:** 2026-08-13

**Authors:** Jiang Nan, Wu ZuXia, Zhao XiaoMin, Tang XueMei, Jiang ShanWen, Cheng Qing, Tu MeiJuan

**Affiliations:** Division of Life Sciences and Medicine, Department of Hematology, The First Affiliated Hospital of USTC, University of Science and Technology of China, Hefei, Anhui 230001 China

**Keywords:** Journey map, allo-HSCT, Health management, Symptom management, Quality of life

## Abstract

**Background:**

Allogeneic hematopoietic stem cell transplantation serves as a curative therapy for various hematologic disorders. However, existing care models often lack a holistic perspective on patients' entire journey from diagnosis to long-term survival.

**Objective:**

This study employs patient journey mapping to systematically analyze the comprehensive health management needs of allo-HSCT patients throughout their entire treatment cycle.

**Methods:**

We adopted a descriptive qualitative design. Semi-structured interviews were conducted with 16 allo-HSCT patients between November and December 2025. Interview data were analyzed using inductive content analysis. Interview data were analyzed using conventional inductive content analysis.

**Results:**

The patient treatment process was delineated into four distinct phases—screening and evaluation, transplantation, maintenance therapy, and long-term follow-up—with a total of 31 themes identified across three dimensions: task execution, emotional states, and pain point management. The symptom burden shifted dynamically throughout the treatment trajectory: psychological distress and decisional paralysis dominated the screening phase, escalating into concurrent acute physical symptoms during transplantation, and eventually transitioning to persistent functional decline driven by chronic graft-versus-host disease in long-term survivorship. Emotional experiences followed a parallel trajectory—from intense information needs and guilt in the screening phase, to profound isolation and emotional ambivalence during transplantation, and finally to a complex interplay of resilience and helplessness in the face of lasting sequelae during recovery. Across all phases, systemic pain points consistently centered on fragmented care transitions, a persistent misalignment between information provision and patients' evolving needs, a lack of sustainable support for lifelong self-management, and the compounding impact of financial strain and social role loss.

**Conclusion:**

The patient journey map constructed in this study offers a visual framework for understanding the holistic care needs of allo-HSCT patients across the entire trajectory and pinpoints four critical intervention nodes. Based on these findings, we recommend implementing phase-tailored health education aligned with patients' evolving cognitive needs, integrating routine financial toxicity screening into standard care pathways, strengthening structured transitional support at discharge, and establishing long-term survivorship programs that encompass vocational rehabilitation and peer support networks. These evidence-informed strategies can facilitate the transition from fragmented, event-driven care to integrated, patient-centered care across the entire transplant continuum.

## Background

Allogeneic hematopoietic stem cell transplantation(allo-HSCT) stands as a potentially curative therapy for a variety of malignant and non-malignant hematologic disorders, including leukemias, lymphomas, and aplastic anemia [1]. Globally, transplantation activity continues to rise, with the Worldwide Network for Blood and Marrow Transplantation reporting over 50,000 allo-HSCT procedures annually, a number that has steadily increased over the past decade [2]. This growth is mirrored in China, which has emerged as one of the world’s largest transplantation centers. According to the Chinese Blood and Marrow Transplantation Registry, the annual number of allo-HSCTs in China exceeded 15,000 in 2023, demonstrating a rapid expansion of both capacity and clinical expertise [3].

Despite its therapeutic promise, allo-HSCT represents one of the most complex and arduous medical journeys in modern medicine. The process extends far beyond the acute inpatient phase, encompassing a prolonged and unpredictable trajectory from pre-transplant evaluation to long-term survivorship. Patients face a formidable array of challenges, including but not limited to life-threatening complications such as graft-versus-host disease (GVHD), infections due to profound immunosuppression, disease relapse, and multi-organ toxicities [4]. Critically, the burden is not merely physical. A substantial body of international literature highlights the profound psychosocial distress, financial toxicity, and deteriorated health-related quality of life (HRQoL) experienced by allo-HSCT recipients and their caregivers [5, 6]. For instance, studies report that over 30% of long-term survivors experience severe fatigue, and anxiety/depression rates remain significantly higher than in the general population years after transplantation [7].

The management of these multifaceted challenges necessitates continuous, coordinated, and patient-centered care. However, the current healthcare delivery model for allo-HSCT is often fragmented [8, 9]. Care transitions—from tertiary transplant centers back to local communities or primary care—are frequently marked by gaps in communication [10], unclear responsibility delineation [11], and unmet patient needs [12]. This fragmentation can lead to delayed recognition of complications [13], poor adherence to long-term monitoring protocols [14], and ultimately, inferior clinical outcomes and patient experiences [15]. In China, this issue is compounded by regional disparities in healthcare resources and a developing primary care system, making the post-discharge continuum of care a critical vulnerability [16]. By “post-discharge continuum,” we refer to the coordinated sequence of care activities—including medication management, symptom monitoring, complication surveillance, and psychosocial support—that patients require after leaving the transplant center, typically spanning from hospital discharge through the maintenance therapy and long-term follow-up phases.

To address these systemic gaps, there is a growing international emphasis on re-engineering care pathways through patient-centric frameworks. The **patient journey map** has emerged as a valuable qualitative tool in healthcare design and improvement. It moves beyond linear clinical pathways by visually synthesizing the holistic patient experience across time, capturing not only clinical “touchpoints” but also the emotional states, pain points, and unmet needs at each stage [17]. While this methodology has been successfully applied in chronic disease management (e.g., diabetes, cancer) to identify service gaps and design interventions [18, 19], its application in the exceptionally complex and high-stakes context of allo-HSCT remains limited, particularly in the Chinese healthcare setting.

Therefore, this study aims to fill this knowledge gap by employing a patient journey mapping approach to qualitatively explore the comprehensive health management experiences of allo-HSCT patients in China. Specifically, our primary objectives are: (1) to delineate the distinct phases of the allo-HSCT journey from the patient perspective; (2) to identify the key tasks, emotional transitions, and systemic pain points patients encounter at each phase; and (3) to construct an evidence-based patient journey map that can inform the development of targeted, multidisciplinary interventions. The target population is adult allo-HSCT recipients treated at a tertiary hospital in China. Our findings are intended to provide actionable insights for transplant teams—including physicians, nurses, psychologists, social workers, and allied health professionals—to redesign care pathways that better align with patient needs across the entire trajectory.

## Methods

### Study design

This study employs a descriptive qualitative research design, guided by the patient journey map framework, to explore the holistic health management experience of patients undergoing allo-HSCT. The journey map construction utilizes a multi-method, phased strategy integrating the following components: (1) Reviewing clinical practice guidelines to establish distinct clinical phases; (2) Outlining typical clinical pathways through preliminary consultations with multidisciplinary clinical teams (including transplant physicians, specialized nurses); (3) Collecting first-person experiential data from patients via semi-structured in-depth interviews, capturing tasks, emotions, pain points, and unmet needs to refine and enrich the framework.

Allo-HSCT involves critical and prolonged transitions—from pre-transplant evaluation and conditioning, through the acute inpatient Transplant Phase and early recovery, to long-term survivorship and Long-term Follow-up Phase. Each phase is shaped by distinct and evolving clinical, psychological, and social challenges. The chosen qualitative design, utilizing in-depth interviews and inductive content analysis, allowed patients to narrate their lived experiences chronologically and holistically. This method was essential for revealing nuanced insights into symptom burden trajectories, psychological adaptation processes, care coordination gaps, and the dynamic evolution of support needs across this extended continuum. The Consolidated Criteria for Reporting Qualitative Research (COREQ) guidelines were used to evaluate the qualitative research reports in this study.

### Participants

A purposive sampling strategy was employed to recruit participants from the Department of Hematology at a tertiary Grade A hospital in Anhui Province between November and December 2025. The target population comprised adult patients who had undergone allo-HSCT. Inclusion criteria were: (1) age ≥ 18 years; (2) having received allo-HSCT; (3) being conscious and possessing adequate communication and comprehension abilities to participate in an interview; and (4) providing informed consent voluntarily. Exclusion criteria were: (1) comorbid severe dysfunction of major organs (e.g., heart, liver, or renal failure); (2) diagnosed psychiatric disorders or cognitive impairment that would impede cooperation with the study procedures; or (3) experiencing severe post-transplant complications with an estimated life expectancy of ≤ 3 months, as assessed by the treating physician. Sample size was determined based on the principle of data saturation, whereby recruitment continued until no new themes or insights emerged from consecutive interviews [20, 21]. Saturation was confirmed through an iterative monitoring process: after each interview, the two primary coders independently reviewed newly emerged codes and met to discuss whether any novel themes or categories had appeared. Saturation was considered achieved when three consecutive interviews (participants N14–N16) yielded no new codes or themes, and the established thematic framework—organized across the four treatment phases (Screening and Evaluation Phase, Transplant Phase, Maintenance Therapy Phase, and Long-term Follow-up Phase) and three functional dimensions (tasks, emotions, and pain points)—comprehensively captured all aspects of the participant-reported experiences. This determination was further validated through team consensus discussions during peer feedback meetings. This criterion was met after including a total of 16 participants. The demographic and clinical characteristics of the enrolled patients are summarized in Table 1. All participants provided written informed consent after receiving a comprehensive explanation of the study aims, procedures, potential risks, and benefits, as well as their right to withdraw at any time without affecting their standard medical care.

### Research team

Our research team consists of eight members, including one hematologist doctoral supervisor, three head nurses, two oncology nurses, and two master’s-prepared nursing researchers. All members have received formal training in qualitative research methods and possess extensive clinical nursing experience. All interviews were conducted independently by JN, who holds a master’s degree in nursing and has substantial experience in qualitative research. TMJ was responsible for participant recruitment and coordination of interview logistics. After each interview, one researcher performed verbatim transcription and preliminary data processing, while another researcher was responsible for coding verification. To ensure methodological rigor, the supervisors held regular peer review meetings to address any ambiguous interpretations that arose during data analysis. All coding and analysis were independently performed by two researchers, with disagreements resolved through team consensus.

## Research method

### Desk literature review study

The research team conducted a desk literature review to summarize the existing clinical data on patients undergoing allo-HSCT [9, 22]. Through repeated discussions, we developed a timeline of the patient’s journey, identifying four key phases: Screening and Evaluation Phase, Transplant Phase, Maintenance Therapy Phase, and Long-term Follow-up Phase. In the visual chart, key decision points and care transitions were systematically marked.

### Semi-structured interviews

#### Interview guide *semi-structured interview*

##### Interview outline

This interview outline was developed based on literature review and group discussions, and refined following pilot interviews with three patients. Two experts with specialized knowledge in qualitative research and hematology nursing were invited to review the outline, and further modifications were made according to their feedback.

The complete interview guide consisted of the following five open-ended questions, with additional spontaneous probes used flexibly to explore emerging themes.

① At which stage of allogeneic hematopoietic stem cell transplantation are you currently?

② During the treatment process, which uncomfortable experiences left the deepest impression on you? How did these experiences affect your daily life and mental state?

③ When dealing with these uncomfortable experiences, which factors helped you alleviate them? Which factors might have exacerbated the situation?

④ During the symptom management process, what difficulties or setbacks did you encounter? How did you attempt to solve these problems?

⑤ In terms of medical care, which aspects do you most hope that medical staff can provide more support and assistance for you?

### Data collection process

Before the interview, the researchers clearly informed the respondents of the research purpose and signed the informed consent form. After that, they arranged the subsequent interview. The interview location was chosen according to the respondents’ wishes, usually in a quiet place in the department office, and no third parties were allowed to enter. During the interview, the researchers requested the respondents to clarify and confirm when there were any doubtful statements. At the same time, they closely observed the non-verbal reactions. The interview was recorded throughout, and it ended when the information was saturated. Each interview lasted approximately 20–40 min.

### Data analysis

This study employed the seven-step analysis method proposed by Colaizzi for systematic analysis [23]. Interviewers first transcribed the interview content verbatim into Chinese text to preserve participants’ original intent and nuances. Researchers then familiarized themselves with the data through repeated reading of the transcripts to gain an in-depth understanding of the content’s implications. Two researchers independently identified meaningful text units and generated initial codes, subsequently reaching coding consensus through comparative discussion; disagreements were resolved through team consensus, ensuring investigator triangulation. Codes were organized and categorized according to a predefined framework encompassing three dimensions: tasks, emotions, and pain points. Researchers then examined the relationships between categories and subcategories to optimize the classification structure. Through iterative analysis, data were continuously compared and integrated until no new categories emerged and data saturation was achieved; this criterion was met upon inclusion of the 16th participant. Finally, the journey map framework was integrated with the interview data for result interpretation and visualization, intuitively illustrating participants’ dynamic health management needs throughout the treatment trajectory. The finalized patient journey map underwent member checking with participants to confirm the accuracy and completeness of the findings.

### Ethical considerations

This study was conducted in accordance with the Declaration of Helsinki. This study was approved by the Medical Research Ethics Committee of the First Affiliated Hospital of USTC (Approval Number: 2025KY伦审第503号). Before data collection, all participants gave written informed consent, including permission for audio recording. Participants retained the right to withdraw from the interview at any time without penalty, although none did so. To ensure the privacy and data confidentiality of the participants throughout the entire research process, during the transcription and data analysis stages, all identifiable information of the participants was deleted and replaced with unique research codes (e.g., N1, N2). All digital records (audio recordings and field notes) were stored under this anonymous code. Furthermore, all electronic data were stored on a secure and protected hard drive.

### Rigour and reflexivity

To ensure the credibility of this research, multiple rigorous measures were implemented. Credibility was enhanced through peer review and member validation, confirming that the findings accurately reflect participants’ perspectives. The original Chinese records were reviewed by the first author and verified for linguistic fluency by the second author, thereby strengthening reliability. Emerging codes, categories, and subcategories were translated into English, with discrepancies resolved through group discussions. To maintain consistency and accuracy in coding and analysis, the final translation underwent comprehensive cross-checking against the original text. The final categories and subcategories are presented bilingually in Chinese and English, accompanied by participant quotations. This provides contextualized evidence for the health management needs of hematopoietic stem cell transplant patients, ensuring verifiability.

## Results

### Characteristics of participants

This study included 16 patients who underwent allogeneic hematopoietic stem cell transplantation, with a mean age of 35.38 ± 12.18 years (range 19–57 years). The cohort comprised 7 females and 9 males, with 75% of patients being married. Detailed information is presented in Table 1.

**Table 1:** General characteristics of the patients (*n* = 16)

ID	Gender	Age (years)	Education	Marital Status	Diagnosis	Transplant Method	Post-Transplant (months)
N1	Female	40	Junior High School	Married	Severe Aplastic Anemia	Umbilical Cord Blood	6
N2	Male	19	Junior High School	Unmarried	Acute Lymphoblastic Leukemia	Umbilical Cord Blood	4
N3	Female	57	Primary School	Married	Acute Myeloid Leukemia	Umbilical Cord Blood	24
N4	Female	36	Junior High School	Married	Aplastic Anemia	Peripheral Blood Stem Cell	25
N5	Male	22	Associate Degree	Unmarried	Acute Myeloid Leukemia	Umbilical Cord Blood	26
N6	Male	31	Technical Secondary School	Married	Acute Lymphoblastic Leukemia	Peripheral Blood Stem Cell	30
N7	Male	35	Associate Degree	Married	Acute Myeloid Leukemia	Umbilical Cord Blood	26
N8	Male	22	Junior High School	Unmarried	Acute Lymphoblastic Leukemia	Umbilical Cord Blood	4
N9	Female	55	Junior High School	Married	Myelodysplastic Syndrome	Peripheral Blood Stem Cell	4
N10	Male	21	High School	Unmarried	Acute Myeloid Leukemia	Umbilical Cord Blood	3
N11	Female	37	Associate Degree	Married	Myelodysplastic Syndrome	Peripheral Blood Stem Cell	10
N12	Male	37	Junior High School	Married	Acute Lymphoblastic Leukemia	Umbilical Cord Blood	3
N13	Female	46	Bachelor’s Degree	Married	Myelodysplastic Syndrome	Umbilical Cord Blood	11
N14	Female	51	Junior High School	Married	Acute Myeloid Leukemia	Umbilical Cord Blood	5
N15	Male	24	Junior High School	Married	Severe Aplastic Anemia	Peripheral Blood Stem Cell	3
N16	Male	33	Bachelor’s Degree	Married	Severe Aplastic Anemia	Umbilical Cord Blood	12

Note: Post-Transplant (months) indicates the time elapsed since the date of hematopoietic stem cell reinfusion (Day 0 of transplantation), not since hospital discharge

### Construction of the journey framework

**Fig. 1:**
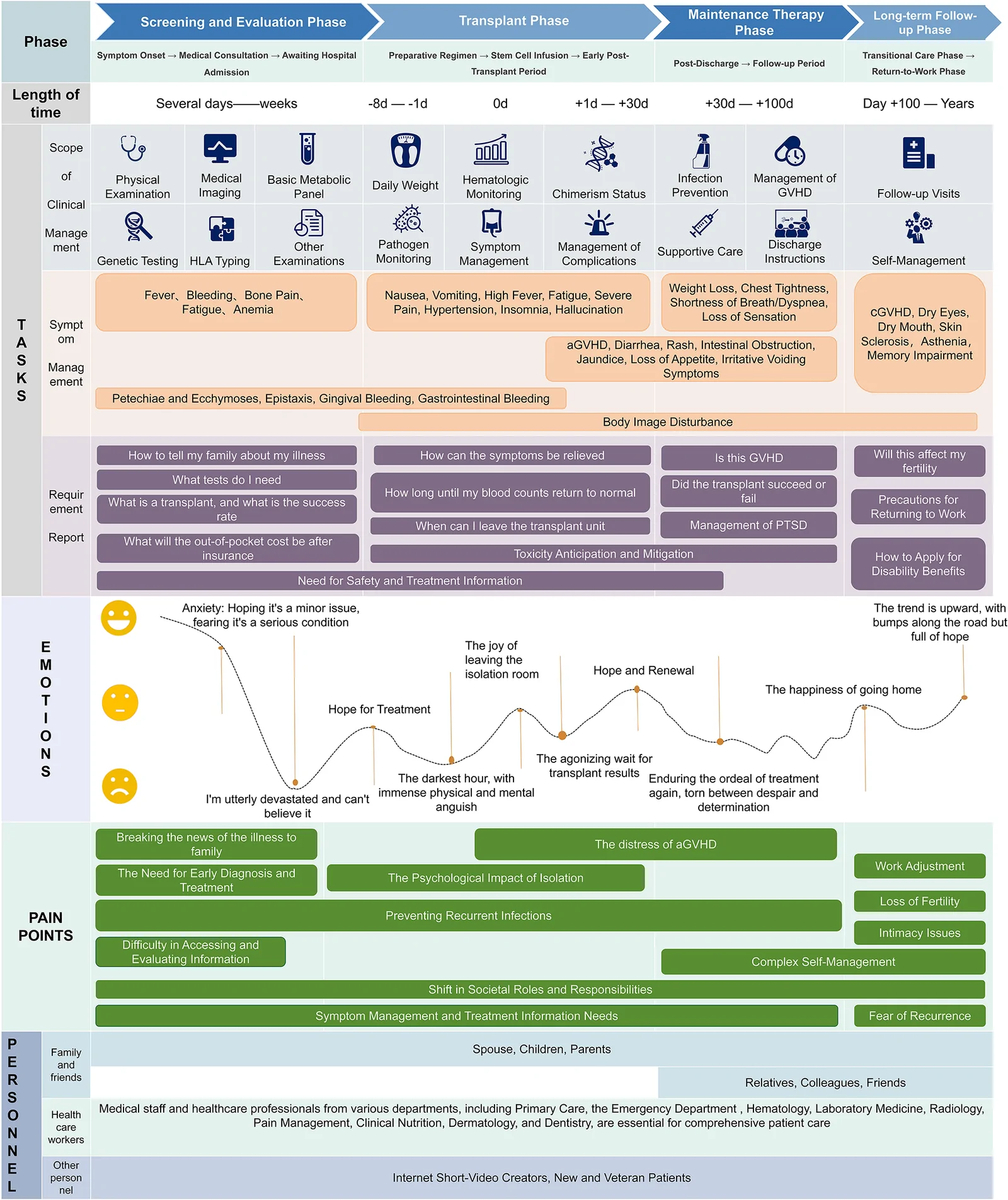
Health management journey map for allo-HSCT patients

The patient journey map adopts a dual-axis structure: the timeline (horizontal axis) and the functional dimension (vertical axis). Under the guidance of the consensus of clinical experts, we divided the timeline of allogeneic hematopoietic stem cell transplant patients into four distinct phases: Screening and Evaluation Phase, Transplant Phase, Maintenance Therapy Phase, and Long-term Follow-up Phase. The vertical axis includes three functional domains that are crucial for the health management trajectory: tasks, emotions, and pain points. Through strict coding, 31 themes were merged, systematically documenting the behavioral adaptation, emotional changes, and care barriers in all health management stages. This process achieved the construction of a multi-dimensional storyline, ultimately forming a comprehensive health management journey map for allogeneic hematopoietic stem cell transplant patients (Fig. 1). Figure 1 visually presents the health management journey map for allo-HSCT patients, with the four treatment phases displayed along the horizontal axis and the three functional dimensions (tasks, emotions, and pain points) along the vertical axis, color-coded to illustrate key tasks, emotional transitions, and systemic barriers at each stage.

### Screening and evaluation phase

#### Tasks

① Symptom Identification and Diagnosis

Patients’ early symptoms are not obvious, such as fever and a few bruises, which are easily confused with the flu or accidental bumps. N2 (4 months post-transplant): “After taking cold medicine for three days, the fever didn’t subside. I went to a small hospital to check my blood, and the doctor saw the report and changed his expression immediately. He told me to go to a big hospital immediately.” N8 (4 months post-transplant): “At first, I felt tired. When I got home from work, I just wanted to lie down. I thought it was because I was too busy and didn’t rest well. Later, my legs turned blue and purple for no reason, and I went for a check-up.” N9 (4 months post-transplant): “I had toothache for three days, but the gum kept bleeding despite the dental cleaning. When I checked my platelets, it was only six. That night, I was hospitalized.”

② Joint Decision-Making

The decision-making process is a combination of clinical evidence, patient preferences, and professional knowledge. N10 (3 months post-transplant): “The doctor talked a lot with us, reviewed the data, understood the risks, and I also made up my mind. I still decided to try the transplant once.” N14 (5 months post-transplant): “At first, I thought my physical condition might not be able to withstand rejection and wanted to choose conservative treatment to ensure my quality of life.”

#### Emotions

① Information Hunger

Under the emotions of anxiety and panic, patients have an extreme desire for information. N11 (10 months post-transplant): “When waiting for the key results of bone marrow puncture, my phone rang and I felt scared. I was both expecting and afraid to hear the news from the hospital.” N13 (11 months post-transplant): “I couldn’t focus on my work at all. I searched for medical terms frantically and knew it was wrong, but I habitually matched them to the situation.”

② Hope and Despair Coexisting

The uncertainty of treatment intensified the patient’s sense of despair, while the feasibility of radical treatment injected hope. These two contradictory emotions did not simply alternate but presented a coexisting game state. N5 (26 months post-transplant): “When I was diagnosed, it felt like a bolt from the blue. How could it be me! Before, I was doing fine. Logically, I knew I should believe in science, but emotionally, I couldn’t accept the reality.” N2 (4 months post-transplant): “Transplant is the only way to cure it. In my opinion, it’s ‘saving oneself by killing oneself.’ I really want to survive, but I’m so afraid of the treatment methods that I want to escape.”

③ Guilt

Patients felt that they had become a heavy burden for their families and that the treatment would deplete their family’s savings and deprive them of their role as an economic contributor, causing them to feel guilty. N11 (10 months post-transplant): “My husband had an opportunity for promotion, but he gave it up without hesitation to accompany me for treatment. Seeing him tired and thin, I felt like a knife was cutting me.” N15 (3 months post-transplant):“Watching my mother draw my bone marrow, it should have been her time to enjoy her old age, but she had to suffer for me.” N14 (5 months post-transplant): “The huge medical expenses were like an endless pit. The family’s savings were almost depleted. If I couldn’t survive in the end, wouldn’t it be a total loss for the family, leaving them with a mountain of debts?”

#### Pain points

① Lack of Health Literacy

The early symptoms of blood diseases are easily overlooked, and patients lack the correct identification of symptoms. N16 (12 months post-transplant): “I thought it was just a result of work fatigue and immunity decline. It was several months before I was diagnosed with leukemia.” N12 (3 months post-transplant): “After searching online, I self-diagnosed as nutritional anemia and only ate dates and spinach on my own. I didn’t go to the hospital in time.”

② Efficiency of Medical Treatment Process

The diagnosis of this disease relies heavily on advanced medical centers. Examinations mostly adopt linear appointments rather than integrated arrangements, and the recognition of report results is limited. Medical expenses increase. N3 (24 months post-transplant): “I live in a remote town. The local hospital couldn’t perform key examinations such as bone marrow puncture.” N1 (6 months post-transplant): “The hospital doesn’t recognize reports from non-local hospitals, and some examinations can only be repeated.” N2 (4 months post-transplant): “The doctor also issued an admission notice, but there was no bed, and I didn’t have immunity. I didn’t know when I could get a bed for treatment.”

③ Decision Paralysis

The negative emotions at the beginning of the diagnosis reduced the patient’s information processing efficiency, and the significant differences in treatment outcomes of different patients made them confused in contradictory information. N4 (25 months post-transplant): “The doctor presented me with two options, explaining their respective success rates and risks. But my mind was in a complete mess. Looking at the thick stack of materials, I couldn’t even read a single word.” N7 (26 months post-transplant): “I did some research online. Doctor A said transplantation was the only hope, while Expert B suggested trying targeted drugs first. On the forum, some said transplantation was good, while others said they would rather die due to severe rejection. The more I read, the more confused I became about what to do.” N10 (3 months post-transplant): “Finally, I couldn’t bear this mental torture anymore. I told the doctor, ‘Please don’t make me choose. Do whatever you think is best. I’m entrusting my life to you.‘”.

### Transplant phase

#### Tasks

① Symptom management challenges

Patients simultaneously experience various symptoms such as mucositis (oral and digestive tract), severe nausea and vomiting, diarrhea, rashes, etc. These symptoms exacerbate each other, forming a vicious cycle. N9 (4 months post-transplant): “The rash area is itchy and painful. I can’t help but want to scratch, but as soon as I turn over or move, I feel dizzy and want to vomit. When I vomit, my muscles tense up, and when I exert force on my stomach, the diarrhea becomes more severe.” N12 (3 months post-transplant)(pained expression): “My mouth is burning, my stomach is churning, my intestines are spasming, my skin is stinging, and my entire body is completely drained.”

② Establishment of care support system

In an absolutely isolated treatment environment, the medical team has established a comprehensive life support system that integrates medical monitoring, daily care, and emotional support. N8 (4 months post-transplant): “At that time, I didn’t even have the strength to turn over. The nurses not only helped me handle the most private matters but also always comforted me softly. It was their professionalism and kindness that maintained my dignity.” N15 (3 months post-transplant):“Every day, I most looked forward to hearing the doctor’s blood test results for today. Even a rise of 0.1 was a hope.”

#### Emotions

① Expectation

It is a kind of adaptive psychology of patients [24], which helps them shift from anxiety to focusing on the core goal of cooperating with treatment. N1 (6 months post-transplant): “At that time, I didn’t think about anything else. I just wanted to enter the cabin, receive reinfusion, and increase my cells.” N15 (3 months post-transplant):“I repeatedly checked the list of things to bring when entering the cabin and made thorough preparations.” N14 (5 months post-transplant): “I thought that when I came out of the cabin, I would leave behind all the pain of these years and everything would be a new beginning.”

② Loneliness

It stems from physical isolation, the inability to share emotional experiences, and the loss of control over life. N15 (3 months post-transplant):“Everyone is living their lives, but only my time has come to a standstill. I am isolated from the world, and it is more tormenting than the pain.” N9 (4 months post-transplant): “The white blood cell count rose in the morning, I was happy; in the afternoon, the platelet count dropped again, I was disappointed. My emotions were completely controlled by these numbers. When will the cells grow back? Will they grow again?” N11 (10 months post-transplant): “Sometimes I was so miserable that I didn’t want to live anymore, but even the strength to say this was lacking. Silent screams, only you can hear them.”

③ Fear

Fear is often deliberately suppressed, but it is everywhere. N6 (30 months post-transplant): “I said I was full of hope on the surface, but when it was quiet at night, ‘What if the cells don’t grow?’ ‘What if there is a serious infection?’ These thoughts would pop up. I didn’t dare to think too much, but I couldn’t stop thinking about it.“N3 (24 months post-transplant): “I knew that a stranger had given me a second chance at life. I desperately wanted to get better, as if I had failed, I was sorry for that selfless gift.”

#### Pain points

① Complexity of Symptom Management

After undergoing pre-treatment and transplantation, patients often experience multiple symptoms such as mucositis, severe gastrointestinal reactions, rashes, and infections. N9 (4 months post-transplant): “Vomiting and diarrhea occur simultaneously, and it’s impossible to control. The skin rashes become even itchier when fever occurs, and the various symptoms are mixed together, making it difficult to determine which part is more painful.” N12 (3 months post-transplant): “The oral and esophageal mucosa is severely ulcerated, and nothing can be eaten, including liquid nutritional solutions. The weight drops rapidly, and I feel extremely weak.”

② Dual Suppression of Emotional Expression

On the one hand, positive and optimistic emotions are encouraged by the medical environment, but this may cause patients to refrain from expressing their true emotions due to fear of being “not strong enough.” On the other hand, negative emotions such as despair and anger are often concealed by patients, fearing being regarded as “not cooperating with treatment” or increasing the burden on their families. N6 (30 months post-transplant): “Even when communicating with family members by voice, I say I’m fine, but in reality, I often feel uneasy.” N10 (3 months post-transplant): “For a period of time, my mood was extremely low, and I dared not tell the medical staff, fearing that it would affect their judgment of the treatment effect.”

③ Systemic Interruption of Social Connection

The absolute isolation of the transplant cabin has forcibly separated the patient’s original social relationship network [25]. This disconnection also sets up obstacles for the patient’s reintegration into society after the treatment. N15 (3 months post-transplant):“Completely isolated from the outside world within the cabin, I can only learn about the outside world through my mobile phone.” N13 (11 months post-transplant): “During the treatment, I couldn’t fulfill my parental duties, feeling guilty for affecting the parent-child relationship, and worried that it would be affected.”

### Maintenance therapy phase

#### Tasks

① Health Monitoring

This includes regular follow-ups and long-term management of chronic graft-versus-host disease (cGVHD), among other things. N2 (4 months post-transplant): “The day before the re-examination, I would organize all the medical records and checklists in order.” N10 (3 months post-transplant): “I set three alarms every day to remind myself to take anti-rejection medication.” N13 (11 months post-transplant): “There is always disinfectant at the entrance of my home. If I have to go to crowded places, I not only wear a mask but also try to avoid standing in crowded areas.”

② Adjusting to the New Normal

This involves the comprehensive integration of physical, mental, behavioral, and social roles, which is a difficult process of self-reinvention. N11 (10 months post-transplant): “Not only do I have swollen face, curly hair, and various pigmentation on my body, but I can only accept this as part of my life.” N7 (26 months post-transplant): “My sense of taste has changed. I try to have a diverse diet to ensure adequate nutrition.” N6 (30 months post-transplant): “Now I never do anything risky. I don’t dare to stay up late or eat randomly. After rebirth, I realized how precious health is.”

#### Emotions

① Resilience and Acceptance

After experiencing extreme treatment challenges, patients demonstrate remarkable resilience and a profound understanding of life. N1 (6 months post-transplant): “Every time the re-examination results are good, it gives me great encouragement. Living is really not easy.” N5 (26 months post-transplant): “I know there may be changes in the future, but I have learned not to waste energy. Living each present moment well is my best preparation for the future.” N6 (30 months post-transplant): “Before the illness, I was anxious about things like promotion and buying a house. Now I feel that except for life and death, everything else is trivial.”

② Helplessness and Irritation

The symptoms of chronic rejection are fragmented and persistent, requiring patients to adapt their entire lives. N11 (10 months post-transplant): “I could endure intense chemotherapy and the cell-free period, but I was almost tortured to death by this chronic rejection.” N12 (3 months post-transplant): “To suppress rejection, I have to take a lot of immunosuppressants, but these drugs themselves have side effects such as tremors and insomnia. It’s impossible to find a comfortable balance.” N15 (3 months post-transplant):“Dry eyes, bitter mouth, stiff joints. These problems alone don’t seem serious, but they recur repeatedly, and I don’t know what symptoms will appear next.”

③ Loss and Sadness

The illness not only deprived patients of their healthy bodies but also shattered their future blueprint built on health. N14 (5 months post-transplant): “A large amount of hormones caused me to have a full moon face and a buffalo back. Looking in the mirror, it felt like a different person.” N5 (26 months post-transplant): “I used to be able to run and climb mountains. Now I need to take a breath and rest after walking a few steps. I am very disappointed with myself.” N15 (3 months post-transplant):“Before the illness, I had a clear plan for the future. Now none of these can be achieved.”

#### Pain points

① The Heavy Burden of Lifelong Management

Patients need to transform into lifelong highly self-disciplined health managers, with their energy constantly being consumed, and there is no sense of relief from truly recovering. N3 (24 months post-transplant): “Every day, I need to check the details of my body, such as whether there are new rashes on the skin? Any minor changes could be a sign of chronic rejection.” N8 (4 months post-transplant): “Sometimes, I get really fed up with all this. I just want to be selfish for once and have a meal at a hot pot restaurant.” N10 (3 months post-transplant): “Every time I feel something uncomfortable on my body, the first reaction is ‘Is it a recurrence?’ This suspicion of my body may last for my entire life.”

② The Blow of Social Isolation

Patients feel the annihilation of their self-worth and the powerlessness of being disconnected from society. N12 (3 months post-transplant): “Friends on the social media are sharing trips, but my life seems to have been paused. They are going to vast places, while my world is just the few kilometers around the hospital and home.” N13 (11 months post-transplant): “When I hear everyone discussing the latest technologies and projects, I am completely at a loss for words. The thing I fear most is not the illness, but that I have become a useless person.”

③ The Impact of Economic Toxicity

It not only manifests in income and expenditure, but also permeates psychological state, treatment decisions, family relationships, and survival outcomes. N16 (12 months post-transplant): “My life was saved, but my financial situation was exhausted.” “Every day of my life is like burning money. This feeling is so painful.” N13 (11 months post-transplant): “The doctor suggested using a new targeted drug. It costs tens of thousands a month, all out of my own pocket. I’m not saying I don’t want to live; it’s just that I really can’t afford it.“N3 (24 months post-transplant): “I often wonder if I’m gone, will my family be able to relax a bit?”

### Long-term follow-up phase

#### Tasks

① Reintegration into Society

After recovery, patients generally face physical functional impairments and need to adjust their bodies to adapt. N9 (4 months post-transplant): “I’m a little worried that my physical condition won’t keep up. I’m doing rehabilitation training every day and hope to return to my previous state.” N16 (12 months post-transplant): “I gathered the courage to tell my colleagues about my situation and informed them that I might not be able to work overtime.”

② Career Transition

Due to physical conditions that cannot withstand the intensity of the original job, some patients actively implement career transitions. N3 (24 months post-transplant): “I have severe numbness in both lower limbs. I got a disability certificate and worked as a craftsman in a factory.” N10 (3 months post-transplant): “I started learning graphic design at home and took on some scattered jobs. Making money is not the primary goal; the important thing is to prove that I can still create value.”

#### Emotions

① Dual Worry

Patients are in a difficult choice between work and health. N5 (26 months post-transplant): “My body can no longer withstand the depletion of high-intensity work. The salary I receive is not enough to cover my medical expenses.” N7 (26 months post-transplant): “If I don’t go to work, what can I expect to earn to take medicine? If I have a recurrence, I really won’t be able to afford hospitalization.”

② Desire for Normal Life

During the rehabilitation process, patients both desire to return to a normal life and fear that progress will stagnate. N8 (4 months post-transplant)(happy): “Today, I made a shot in basketball! Although it was just standing to shoot, it felt like I was back to my previous state.” N11 (10 months post-transplant): “After six months of rehabilitation, the progress has been getting slower. The physiotherapist said not to rush, but can I not rush? I’m afraid I’ll be like this for the rest of my life.”

③ Gratitude for Life

Patients, while accepting the gifts of life, also mourn the precious time taken away by the disease. N9 (4 months post-transplant): “My hair grew back, and it’s all white. That’s good. I don’t have to dye my hair anymore. Living one day is earning one day.” N4 (25 months post-transplant): “I’m grateful to the doctor for saving me, but I also hate this disease that took away my most precious years.”

#### Pain points

① Lack of Understanding Due to Invisible Disability

Patients seem to have recovered physically, but they continue to endure functional impairments after treatment. N13 (11 months post-transplant): “Every explanation feels like revealing my own scars. Now, unless it’s necessary, I’d rather pretend everything is fine. At least this way, others can still look at me with normal eyes.” N15 (3 months post-transplant):“When I was discharged, the doctor said ‘The recovery is good,’ but no one told me how to prove to the company that I can only work for four hours a day. I went to ask for leave with the discharge summary, but the HR said ‘This doesn’t state the working time limit.‘”.

② Risks Excluded from Welfare Support Systems

The existing social security and employment support systems have coverage gaps for this special group of people who are neither completely healthy nor traditional disabled. N16 (12 months post-transplant): “I want to apply for a disability position, but I’m physically intact. The criteria don’t include this item of hematopoietic stem cell transplantation, and the disability certificate can’t be issued. N2 (4 months post-transplant): “The reimbursement for major illness insurance has a cap. The part over the limit is suffocating the entire family. We haven’t considered our special consumption structure where taking medicine is more expensive than eating.”

## Discussion

### Continuity of care needs revealed through a whole-cycle journey perspective in allo-HSCT

Using a patient journey mapping approach, this study systematically examined the full health management trajectory of patients undergoing allogeneic hematopoietic stem cell transplantation (allo-HSCT). The findings demonstrate that patients experience substantial heterogeneity in task demands, emotional responses, and care needs across the four stages of screening and evaluation, transplantation, maintenance therapy, and long-term follow-up. Importantly, despite this stage-specific variability, a continuity of needs persists across the care continuum. These findings suggest that allo-HSCT care is inherently a longitudinal, cross-temporal process. Nevertheless, the current healthcare delivery model is largely episode-based—patients receive intensive care during hospitalization, but support drops off sharply after discharge, leaving them to manage the transition home with little continuity. This finding is consistent with previous studies reporting insufficient transitional care and fragmented continuity in HSCT populations. For instance, a North American survey of 77 HCT programs found that 100% of respondents agreed that complications can arise when survivors transition to remote areas lacking HSCT expertise, and 97% recognized similar risks when pediatric and elderly survivors transition to non-specialist care settings [26]. This discontinuity arises from a fundamental mismatch: patients’ conditions evolve continuously across phases, yet the healthcare system operates in discrete silos—hospitalization, discharge, follow-up—each managed separately [27]. Patients need seamless, continuous care, but what they receive is fragmented, episode-based service—a gap that prevents the smooth transfer of knowledge, skills, and support systems across care settings [28]. Therefore, the value of patient journey mapping lies not only in visualizing the complete care experience, but more importantly in identifying hidden discontinuities within care transitions and providing an evidence-based foundation for developing integrated, continuity-oriented care models. These findings resonate with prior work on transition readiness in HSCT, while extending it by pinpointing decision-to-admission and discharge-to-home as specific nodes where support is most critically needed. It is important to contextualize our findings within the characteristics of our study population. Our cohort comprised patients aged 19–57 years, with five participants (31.25%) aged ≥ 40 years and the oldest at 57 years, reflecting the real-world demographic distribution of allo-HSCT recipients, as transplantation is preferentially indicated for younger patients with better performance status and fewer comorbidities. Regarding disease types, our sample covered the major indications for allo-HSCT—including aplastic anemia, acute lymphoblastic leukemia, acute myeloid leukemia, and MDS—with malignant hematologic disorders accounting for 75% of cases. Notably, 75% of our participants received umbilical cord blood transplantation, which remains a widely used graft source in China. While cord blood transplantation entails specific challenges such as delayed engraftment and slower immune reconstitution, the diversity of disease types and age range in our sample nonetheless captures the core challenges common across allo-HSCT recipients. The identified discontinuities in care transitions suggest the need for structured navigational support. Nurse navigation and case management models have demonstrated benefits in improving care coordination and patient outcomes in oncology settings [29, 30]. However, evidence specifically evaluating such models in allogeneic HSCT populations remains limited. Our findings extend this evidence by identifying the specific transition points—decision-to-admission, discharge-to-home—where navigational support is most critically needed, providing an empirical basis for developing and testing transplant-specific navigation interventions.

### Dynamic evolution of symptom burden and psychosocial trajectories

Consistent with previous studies, this research confirms that allo-HSCT patients experience substantial physical and psychological symptom burden throughout the entire treatment course. However, prior cross-sectional studies have been unable to capture how this burden evolves across time. The present journey-based analysis provides a longitudinal perspective, demonstrating that symptom burden shifts dynamically across different stages of treatment—with each stage presenting distinct care priorities. During the screening and evaluation stage, patients’ primary distress arises from uncertainty regarding treatment eligibility, success probability, and post-transplant outcomes. This uncertainty is driven by information asymmetry, which prevents patients from forming stable expectations and leads to decisional hesitation and repeated confirmation-seeking behaviors (e.g., N7: “the more I read, the more confused I become”). Therefore, early clinical communication should adopt stepwise information delivery and visualized explanations to facilitate comprehension and reduce psychological distress. It is worth noting that while medical teams make substantial efforts to prepare patients, a degree of unpreparedness may be inevitable given the complexity and unpredictability of the allo-HSCT trajectory. Our findings suggest that current preparation efforts are often fragmented and information-dense rather than staged and reinforced over time, which may contribute to patients’ persistent sense of being unprepared. This observation aligns with previous research on information needs in cancer populations, where single-session education has been shown to be insufficient for complex treatment decisions. During the transplantation phase, acute physical symptoms combined with strict protective isolation constitute the dominant stressors. These two factors reinforce each other: physical discomfort is exacerbated by lack of social support, while feelings of isolation intensify symptom perception. Accordingly, care strategies should integrate somatic and psychological interventions. As reported by our participants, when a patient experiences pain, assessing emotional state concurrently is essential—pain and psychological distress were consistently co-reported (e.g., N9, N12). Similarly, when implementing isolation measures, care teams can proactively facilitate video calls with family members, as participants (e.g., N13, N15) highlighted that maintaining family connection was critical to their emotional wellbeing during isolation. By combining routine symptom assessments with brief emotional check-ins and maintaining family connections through video communication during isolation, both physical and emotional needs can be addressed simultaneously. During long-term follow-up, patients gradually shift their focus toward chronic GVHD management and difficulties in restoring social roles. This aligns with evidence documenting cGVHD as a major driver of long-term morbidity, while our journey analysis reveals that this burden accumulates progressively across phases—a pattern less visible in cross-sectional studies. A key challenge in this phase is the abrupt drop-off in professional support after discharge, which leaves patients feeling disconnected and without adequate guidance (e.g., N12: “others are talking about travel, but my world is limited to the hospital and its surroundings”). Effective continuity of care in this phase should therefore emphasize the development of structured self-monitoring frameworks, combined with regular follow-up and peer support systems to facilitate gradual restoration of social integration and functional capacity.

### Misalignment between information provision and cognitive needs

The findings indicate that inadequate information support is not simply a matter of insufficient information delivery, but rather a misalignment between how information is provided and what patients need to know at different stages. Fisher et al. [31] found that HSCT caregivers felt “overwhelmed by the breadth of information” prior to transplantation, and despite receiving extensive education during hospitalization, they continued to face “unanticipated challenges” after patients returned home—a finding that vividly reveals the disconnect between information provision and real-world needs. During the decision-making phase, patients face overly complex information delivered without a structured framework for assimilation and deliberation. During hospitalization, information is delivered intensively but without adequate mechanisms for reinforcement and retention, resulting in poor information recall under physical and psychological stress. During long-term follow-up, information becomes fragmented and poorly synchronized with real-life problems, leading to persistent uncertainty and helplessness. Taken together, these findings suggest that the core issue lies in the design of information delivery systems, which remain process-driven rather than cognition-oriented. Information is currently provided as discrete events rather than as a continuous, staged learning process aligned with patients’ decision-making and adaptation trajectories [11]. Accordingly, information support should be restructured as a longitudinal process. This includes staged decomposition of complex information before transplantation, reinforcement learning using teach-back methods during hospitalization, and the establishment of online knowledge repositories after discharge to support timely problem-solving in home-based care. Through such restructuring, information delivery can shift from passive dissemination to active knowledge management. The specific strategies we propose—including staged education, teach-back methods, and technology-enabled knowledge repositories—are supported by evidence from other chronic disease and cancer care settings. Teach-back has been shown to improve patient comprehension and self-management skills in heart failure and diabetes populations [32, 33]. However, the application of these strategies in the allo-HSCT context has not been systematically evaluated. Our journey map provides phase-specific content priorities that can guide the adaptation of these evidence-based educational strategies to the transplant population, an area warranting future implementation research.

### Economic toxicity as an implicit cross-stage burden

Economic toxicity emerged as a pervasive and continuous burden throughout the allo-HSCT trajectory [34]. Unlike symptom burden or informational challenges, economic toxicity often remains underrecognized in clinical practice, despite its sustained influence on patients’ psychological well-being, treatment decisions, and family functioning. During the screening phase, economic toxicity manifests as anticipatory financial anxiety and concerns regarding household savings. During transplantation, this anticipation shifts to immediate financial pressure driven by intensive medical expenses and income loss [35]. During long-term follow-up, it becomes a chronic burden associated with long-term medication costs, routine monitoring, and reduced employability. Importantly, the impact of economic toxicity extends beyond financial strain, influencing emotional stability and treatment decision-making [36]. This aligns with evidence from oncology research highlighting the clinical significance of financial hardship [37]. Therefore, economic toxicity should be reconceptualized as a systemic care issue rather than an individual financial problem. Integrating economic risk screening into the care pathway, providing timely financial counseling and insurance guidance, and facilitating employment reintegration may help mitigate its long-term impact [38]. To translate these findings into practice, several evidence-based approaches merit consideration. The Comprehensive Score for Financial Toxicity (COST) has been validated in oncology populations as a reliable measure of patient-reported financial distress [39], though its validation in allo-HSCT recipients is still needed. Economic literacy interventions and financial navigation programs have shown preliminary effectiveness in reducing financial burden in cancer patients [40], but evidence in the transplant setting is scarce. Importantly, the quality metrics we propose—such as economic toxicity mitigation rates and patients’ perception of economic burden—are not established standards but rather proposals derived from our empirical findings. We recommend their formal validation through future implementation research before broader adoption. Our study provides the foundational evidence that economic toxicity is a pervasive concern across all phases of the HSCT journey, supporting the need for such future work.

### Social role disruption and reconstruction across the care continuum

Social role disruption represents another persistent cross-cutting theme throughout the allo-HSCT journey. Patients experience progressive loss of social roles from diagnosis onward—beginning with loss of economic provider identity and family responsibilities, and extending to occupational roles during survivorship [41]. During transplantation, isolation measures further sever social connections, while long-term survivorship is characterized by difficulties in occupational reintegration and reduced social participation. Collectively, these findings suggest that social role disruption is not merely a post-treatment phenomenon but a continuously evolving process that begins at diagnosis. The underlying mechanism lies in the historical focus of transplantation care on survival outcomes, with social functioning often assumed to recover spontaneously after physical recovery [42]. However, our findings indicate that social role reconstruction requires structured, multidisciplinary support. A three-dimensional support framework is therefore proposed, integrating medical, rehabilitation, and social domains—medical follow-up with functional assessment, rehabilitation to gradually rebuild physical capacity, and social services to support vocational counseling and reintegration [43]. Notably, individualized adaptation is required across age groups, as younger patients tend to prioritize career restoration, whereas older patients focus more on family role recovery and financial stability. These recommendations are grounded in our empirical findings and existing literature, but their feasibility and effectiveness require further evaluation through implementation research. Although our study focused on patients, these narratives reflect the interdependent patient-caregiver strain well described in prior studies—suggesting that future journey maps should incorporate caregiver perspectives. Our phase-specific findings complement existing HRQoL research by revealing not only that quality of life is impaired, but when and why specific challenges emerge across the survivorship trajectory.

### Integrative interpretation and theoretical implications

This study contributes to the understanding of allo-HSCT care in three key dimensions. Theoretically, it reframes transplant care from a series of hospitalizations to a longitudinal journey from diagnosis through long-term survivorship, emphasizing the need to think beyond individual episodes. Methodologically, it integrates patient-reported emotional experiences with clinical care tasks, enabling the identification of actionable gaps in care delivery. Practically, it highlights critical discontinuities in decision-to-admission transitions, discharge-to-home transitions, information flow, economic burden, and social support systems, providing a structured framework for service optimization and quality improvement.

## Limitations

This study has several limitations. First, the single-center design may limit the generalizability of the findings across different healthcare settings. Second, recall bias may exist due to the retrospective nature of the interviews; however, this was partially mitigated by including patients at varying post-transplant time points. Third, the absence of caregiver and healthcare provider perspectives limits the comprehensiveness of the findings; future studies should incorporate multi-stakeholder perspectives to construct a more holistic journey map. Finally, although thematic saturation was achieved, additional themes may emerge in more diverse populations, warranting further validation. We also acknowledge that we did not perform subgroup analyses based on age, and future studies with larger samples are needed to explore whether the journey map differs across age subgroups.

## Conclusion

In conclusion, this study provides an empirical patient journey map that visualizes the multidimensional and longitudinal challenges allo-HSCT patients face across the care continuum. Current care systems address patient needs primarily through episodic clinical encounters during hospitalization and routine follow-up schedules—approaches that are fragmented and event-driven rather than continuous and patient-centered. Our journey map identifies four precise gaps in current care delivery: (1) lack of systematic transitional support between decision-to-admission and discharge-to-home transitions; (2) information provision that is one-time and information-dense rather than staged and reinforced over time; (3) absence of routine screening for financial toxicity; and (4) insufficient support for long-term social reintegration and vocational rehabilitation. These findings offer an empirical foundation for moving toward an integrated, phase-specific, multidisciplinary care model that anticipates phase-specific challenges: psychological preparation before transplant, dignity-preserving care during acute hospitalization with maintained social connections, structured transitional support at discharge with comprehensive caregiver preparation, precise stage-matched health education, and comprehensive survivorship programs addressing persistent burdens including chronic GVHD, fatigue, social reintegration, and economic toxicity. Implementing these evidence-informed improvements would align healthcare practice with patient needs and ultimately fulfill the promise of transplantation: not merely extending life, but enabling a life worth living. 

## Data Availability

The datasets used and/or analysed during the current study are available from the corresponding author on reasonable request.

## Abbreviations

HSCT: Hematopoietic Stem Cell Transplantation
allo-HSCT: Allogeneic hematopoietic stem cell transplantation
GVHD: Graft-versus-host disease
HRQoL: Health-related quality of life
COREQ: Consolidated Criteria for Reporting Qualitative Research
